# A comprehensive reproductive health program for vulnerable adolescent girls

**DOI:** 10.1186/s12978-020-0866-7

**Published:** 2020-01-23

**Authors:** Razieh Pourkazemi, Mojgan Janighorban, Zahra Boroumandfar, Firoozeh Mostafavi

**Affiliations:** 1 School of Nursing and Midwifery, Isfahan University of Medical Sciences, Isfahan, Iran; 2 Department of reproductive health and Midwifery, Nursing and Midwifery care Research Center, School of Nursing and Midwifery, Isfahan University of Medical Sciences, Isfahan, Iran; 3 Department of health education and promotion, Faculty of health, Isfahan University of Medical Sciences, Isfahan, Iran

**Keywords:** Reproductive and sexual health, Adolescent health services, Vulnerable adolescent girls

## Abstract

**Background:**

Reproductive health of vulnerable adolescent girls is a top priority in global programs. Alcohol consumption, drug abuse, high risk sexual behaviors, sexually transmitted diseases, sexual assault, escape from home, unrestrained sex in the family, history of robbery, imprisonment and living in drug hangouts expose adolescents to different sorts of damage and injury. These adolescent girls are at risk of AIDS and other STDs, unwanted pregnancies, illegal and unsafe abortions, unplanned pregnancy and childbirth, and unsafe motherhood. Therefore, assessing these girls’ reproductive health needs and designing programs to improve their sexual and reproductive health seem to be essential. This study will be conducted to design a comprehensive program for improving the reproductive health of vulnerable adolescent girls.

**Methods:**

The present study is an exploratory sequential mixed methods study (Qual-Quan) designed in three phases. In the first phase, a qualitative study will be used to describe the reproductive health needs of vulnerable adolescent girls, identify facilitating and inhibiting factors, and explain the strategies of reproductive health programs for these girls. Participants will be selected in this phase using purposive sampling method, and the data will be collected through semi-structured interviews. The obtained data will be analyzed using conventional qualitative content analysis. In the second phase, through a quantitative study, the strategies obtained from the qualitative study and review of the literature will be provided to reproductive health care providers, experts, policymakers, and planners to prioritize and select the best strategies. In the third phase, the initial draft of the program will be formulated based on prioritized strategies and will be proposed in a panel comprised of specialists in the areas of reproductive and sexual health, health promotion, social injuries and a psychiatrist. Finally, the final program will be developed and presented after obtaining the agreement and approval of the panel members.

**Discussion:**

Designing a program based on a qualitative study, review of the existing evidence and programs, and using the opinions of experts in different areas can lead to different aspects of reproductive and sexual health of vulnerable adolescent girls. On the other hand, taking into account all cultural sensitivities and taboos as well as political, economic and social barriers, the development of such a program can provide the appropriate possibility of presenting comprehensive reproductive and sexual health services to vulnerable adolescent girls and achieve the goals agreed universally.

## Plain English summary

One of the most important components of human health is reproductive health that planning to provide it, especially with particular regard to women and girls, is one of the essential steps in ensuring the health of the family and society. This study can provide strong, comprehensive information and need-based strategies to ensure reproductive health of vulnerable adolescent girls. This study is an exploratory sequential mixed methods design that consists of three phases. A qualitative study is conducted to assess the reproductive health needs of vulnerable adolescent girls, identify facilitating and inhibiting factors, as well as strategies which can help ensure their reproductive health. The participants in this phase will be selected using purposeful sampling method and the data will be collected using semi-structured interviews. The obtained data is analyzed using qualitative content analysis. In the second phase, through a quantitative study and using the SFF method (suitability, feasibility, flexibility), first introduced by Landsberger, the strategies obtained from the qualitative study and review of the literature will be provided to reproductive health care providers, experts, policymakers, and planners to prioritize and select the best strategies. At the end of this study, the most prioritized strategies are selected. In the third phase, initial drafts of the program will be formulated based on prioritized strategies and will be proposed in a panel comprised of specialists in the areas of reproductive and sexual health, health promotion, social injuries as well as a psychiatrist. Finally, the final program will be developed and presented after obtaining the agreement and approval of the panel members.

Designing a program based on a qualitative study, review of the existing evidence and programs, together with using the opinions of experts in different areas can lead to different aspects of reproductive and sexual health of vulnerable adolescent girls. Furthermore, the development of such a program through considering all cultural sensitivities and taboos as well as political, economic and social barriers, can provide the appropriate possibility of presenting comprehensive reproductive and sexual health services to vulnerable adolescent girls and help us in achieving the internationally agreed goals.

## Background

Reproductive health is one of the most important components of human health. The most important components of reproductive health include informing, educating and counseling on sex and creation of responsibility in both sexes with respect to the cultural conditions of the society, counseling,education and treatment of sexually transmitted infections and sexually transmitted diseases especially AIDS and hepatitis, timely and appropriate prevention and treatment of the complications of abortion, family planning and provision of services in this regard, prevention and appropriate treatment of infertility, provision of prenatal care, safe delivery and postpartum care, especially education and promotion of breastfeeding. The development of programs related to reproductive health and addressing its various components and dimensions at national and international levels is one of the essential steps in ensuring society and family health (with a special focus on women’s and girls’ health) [1]. Adolescents are the most important target group in reproductive health programs [2] Additionally, more than other age groups, adolescents are more susceptible to substance abuse, smoking, alcohol, and engaging in risky behaviors, including unsafe sexual relationships [3]. These factors make adolescents vulnerable to numerous problems including infections, sexually transmitted diseases, especially HIV and hepatitis [4], pelvic inflammatory disease, infertility, ectopic pregnancy [5, 6], unwanted pregnancies, illegal and unsafe abortion [7], unsafe deliveries, maternal and neonatal morbidity and mortality as well as unsafe motherhood [8]. These events affect not only the individual but also the family and society.

It is for these reasons that the provision and promotion of reproductive health among adolescents is one of the most important commitments of societies in achieving Sustainable Development Goals (SDGs), and there is an emphasis on the public access to reproductive health care services and its integration into strategies and programs by 2030 [9].

However, there are still barriers in many countries, especially developing countries, to the design and implementation of reproductive health programs for adolescents. In many cultures, providing reproductive health to this social group faces many challenges [10]. At the political level, the provision of reproductive health to adolescents is of low priority [11] and there are often limited policies and instructions for protection of this right. For example, according to the laws and policies of many countries, the provision of contraceptives for unmarried adolescents is prohibited [12]. Religious beliefs [11], ethical challenges, economic problems, cultural taboos, negative social attitudes [13], as well as a misunderstanding of such programs [10] are among factors leading to the formation of an inhibitory environment for discussion about and provision of reproductive health services especially for vulnerable girls [14]. On the other hand, families, health systems and their staff can also play a major role in preventing the access of vulnerable adolescents to reproductive health through their adverse policies and practices [15]. At the individual level, fear, embarrassment, lack of knowledge, misinformation, superstition, stigma, worry [12], lack of insurance coverage, and lack of financial independence [16] can increase the risks of adolescent reproductive health. Lack of reporting systems on issues such as premarital sex, induced abortion [12], sexual abuse or sexual coercion [17] are also other challenges of providing reproductive health for adolescent.

Therefore, given the necessity of policymaking and program development before doing any operational action, this study was designed through using an exploratory mixed method to identify the reproductive health needs of vulnerable adolescent girls by taking into account all the factors affecting it in the present situation of the society and, then, develop and present a comprehensive reproductive health program for vulnerable adolescent girls.

## Objectives

### Objectives of each phase are as the following

#### Objectives of the first phase: qualitative study

Describe the reproductive health needs of vulnerable adolescent girls.

Identify facilitating and inhibiting of reproductive health factors.

Explain the strategies of reproductive health programs for these girls.

#### Objectives of the second phase: qualitative study

Decision-making and prioritization of the strategies obtained from the qualitative study and review of the literature by using SFF matrix.

#### Objectives of the third phase: the development of the program

The initial draft of the program will be developed based on the prioritized strategies and then modify the program by comments expressed from panel of experts.

## Methods

The present study is an exploratory sequential mixed methods study (Qual-Quan) designed in three phases. The first phase of the study is a qualitative study to describe and evaluate the reproductive health needs of vulnerable adolescent girls, identify facilitating and inhibiting factors, and explain the strategies of reproductive health programs for these girls. In the second phase, through a quantitative study, the strategies obtained from the qualitative study and review of the literature will be provided to reproductive health care providers, experts, policymakers, and planners. The most prioritized strategies will be selected in this phase. In the third phase, the initial draft of the program will be formulated based on the prioritized strategies and will be proposed in a panel consisting of specialists in the areas of reproductive and sexual health, health promotion, social injuries and a psychiatrist. Finally, after obtaining the agreement and approval of the panel members, the final program will be developed and presented.

### First phase: qualitative study

In the first phase of the study, the reproductive health needs of vulnerable adolescent girls, the factors affecting, and the strategies for providing their reproductive health are explained using in-depth semi-structured interviews which will be analyzed using conventional content analysis. The research setting for this study where the adolescent girls will be interviewed includes health homes in welfare centers, prison health centers, the Department of Prevention in the Ministry of Justice, and drug abuse hangouts. Interviews with service providers, decision-makers and policymakers in the areas of vulnerable women and girls will be conducted in their workplaces including public health centers, private offices of the midwives, gynecologists and psychologists, prison, the branch of social injuries in the Ministry of Justice, the Social Security Branch of the Police, the Department of Social Injury in the Department of Education, the Deputy of public health in the province and the university.

### Participation

The population of study in this phase consists of all vulnerable adolescent girls aged between 12 and 19, reproductive health providers, psychologists, decision-makers and policymakers in the field of reproductive health. Purposive sampling method will be used in this phase of the study. Sampling method with the maximum diversity will be used to ensure the validity of the study. As such, adolescent girls with different ages, education levels, and socioeconomic status will be included in the study. Similarly, decision-makers, policymakers, and service providers are also selected to participate in the study, taking into account the maximum diversity in terms of characteristics such as age, education, work experience, type of specialty, job and organizational position. Sampling and data coding will continue until there is no more new code and saturation is reached.

### Inclusion criteria

Inclusion criteria for adolescent girls include aged between 12 and 19 years old, starting and maintaining a menstrual cycle, having no known mental illness that requires to be treated and willingness to participate in the study, and for other participants, willingness to participate in the study.

### Qualitative data collection method

For data collection at this phase, we will use in-depth semi-structured individual interviews, field notes, reminder writing and document review. All actions at this phase will be performed taking into account ethical considerations. Prior to the interviews, the participants will be briefed about the purpose of the research, and written or verbal consent will be obtained from them. The scheduled time for the interview, the duration of the interview and the interview location will be chosen based on the preferences of the participants.

### The method for qualitative data analysis

Conventional qualitative content analysis will be used for data analysis and interpretation.

### Accuracy and reliability of the qualitative data

In order to evaluate the accuracy and stability of the data, the four criteria of credibility, dependability, confirmability and transferability will be used [18].

### Second phase: quantitative study

In this phase, the strategies obtained from the qualitative study and review of the literature will be provided to reproductive health service providers, experts, policymakers and reproductive and sexual health planners for decision-making and prioritization. This phase will be conducted quantitatively and using descriptive research method. In this phase, reviews of the literature will be performed using an extensive and in-depth library study (reference books, theses and research projects) and searching in electronic databases to evaluate the writings related to the subject of study. Searching keywords such as adolescent, vulnerable, unmarried, and reproductive health, all studies published from 2006 to 2019 in -persian or English will be evaluated. For access to this information, PubMed, Science Direct, Web of Science, Cochrane Library, Ovid, Scopus, ProQuest, Magiran, Embase, SID databases will be used. After extracting the strategies, the participants will be provided with a list of strategies in the quantitative phase of the study.

### Research setting

Comprehensive health services centers, the offices of midwives, gynecologists, psychologists and psychiatrists, the Department of health in the province, Social Welfare Organization, the Social Deputy of the Police, the Social Injuries Unit of the Justice Ministry, and the Social Injury Units of the Department of Education are considered as the setting of the study.

### The population of the study

The population of the study consists of the decision-makers and providers of services for vulnerable adolescents include midwives, gynecologists, specialists of reproductive health and health promotion, psychologists, psychiatrists, the authorities of the family health promotion units in the provincial health department, the authorities of social injuries in the police department, the authorities of social injuries in the Ministry of Justice, Social Welfare Organization authorities working in the field of reproductive health, and the authorities of the social injuries unit in the Department of Education.

### Sampling method

Convenience sampling method is used for this part of the study.

### Inclusion criteria

Experts and service providers with the minimum of 2 years of work experience and health professionals and policymakers with at least 3 years of work experience who are willing to participate in the study can take part in it.

### Exclusion criteria

Unwillingness to participate in the study and the questionnaires that are not filled completely.

## Methods

The data collection tool is a two-part questionnaire. The first part deals with the demographic characteristics of the participants, including age, sex, occupation, education, work experience, workplace and job position, and the second part is related to the scoring of the extracted strategies by using SFF matrix. This matrix contains three criteria of importance, necessity and ease of implementation. Each criterion has a score of 1 to 3 (high = 3, average = 2 and low = 1) and, thus, each matrix or strategy can have a score of 3 to 9. The questionnaire of strategies for reproductive health of vulnerable adolescent girls will be completed in-person or via email by the research population identified based on their expertise and activities related to vulnerable adolescents. At this set time, the researcher will refer to these people frequently and follow the work process in answering the questionnaire. In addition, the participants can contact the researcher by telephone if necessary. After the end of the deadline, the questionnaires are collected by the researcher and the scores for each item are calculated and analyzed. At the end of this study, the most prioritized strategies will be used for the development of the program.

### Data analysis method

The collected data will be analyzed by descriptive statistics methods and using SPSS20 statistical software.

### Third phase: the development of the program

In this phase, the initial draft of the program will be developed based on the prioritized strategies. As such, the panel of experts will be used to reach the consensus of experts on the developed draft at this phase. This phase will aim to determine the opinion of experts on the structure and applicability of the program and is performed as a group discussion among experts. In this panel, reproductive health specialists, social injury specialists, psychiatrists, health promotion professionals, and health policymakers and planners are invited, and the draft of the program together with the purpose of the session will be sent to them. Then, the comments expressed in the session, after being approved by all experts, will be used to modify the program.

## Discussion

Vulnerable adolescents are one of the social groups that, because of being affected by poverty, addiction, disability, ethnic and gender discrimination, AIDS, exposure to violence or physical, sexual and psychological abuse by peers or adults in the family, school, or society, child marriage and girl circumcision are more at risk than other adolescents [19]. Various studies have emphasized the importance of adolescent reproductive health as well as the design and implementation of reproductive health programs to improve their reproductive health knowledge, attitude and behaviors and reduce their vulnerability [20–22]. In this regard, the World Health Organization also emphasizes the development of a theoretical and practical model and the development of practical strategies to provide and promote the sexual and reproductive health of adolescents and reduce their risks and vulnerabilities. This action is associated not only with some effects at the individual level but also at the social, economic, and cultural levels [23, 24]. The present study will provide sufficient information and data about the needs, barriers, facilitators, and strategies related to the reproductive health of vulnerable adolescent girls to improve their reproductive and sexual health. The design of a program based on a qualitative study and considering the existing context as well as review of the up-to-date literature and evidence can lead to the overall improvement of reproductive health of vulnerable adolescents and the promotion of health in society. On the other hand, the design and implementation of this program can reduce this vulnerable group’s reproductive health problems such as unwanted pregnancy, unsafe abortion, sexually transmitted infections, AIDS, and, consequently, the costs of treatment and care. In a broader sense, providing and promoting the reproductive and sexual health of these adolescents, this group can be prevented from further vulnerability, while their educational and career opportunities and their quality of life will be improved. We assume that this program has the capacity to be implemented beside other health care promotion programs, and health care providers can use it to provide vulnerable adolescents with comprehensive reproductive health services. With regard to achieving internationally agreed goals in the area of reproductive health, the implementation of the program can improve society’s health indicators such asreduce unwanted pregnancies, unsafe abortions, illnesses and the mortalities & morbidities caused by them. It is our hope that the development and implementation of this program can be another step towards improving the society’s health.

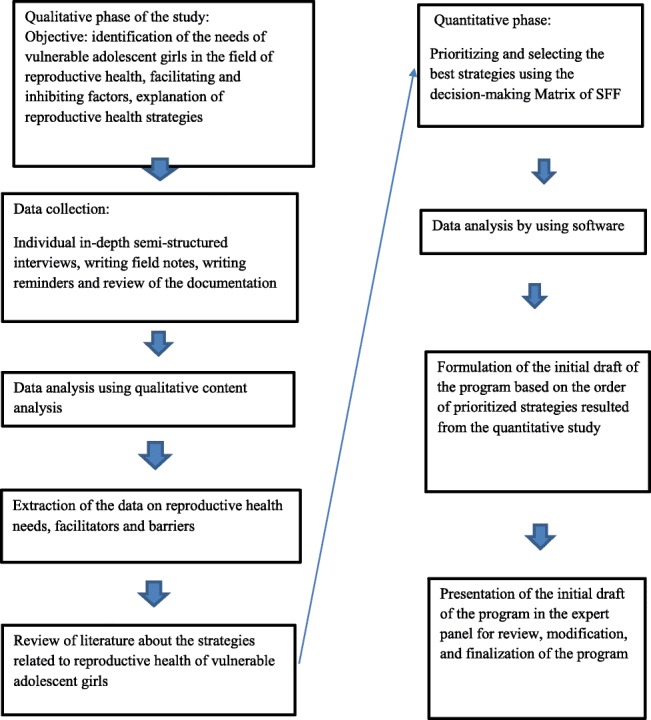



## Data Availability

Not applicable.

## Abbreviation

SFF: Importance, Necessity, Ease of implementation

## References

[1] Hatami H, Razavi M, Eftekhar Ardabily H, Majlesi F (2013). The textbook of public health.

[2] L’Engle KL, Mangone ER, Parcesepe AM, Agarwal S, Ippoliti NB (2016). Mobile phone interventions for adolescent sexual and reproductive health: a systematic review. Pediatrics..

[3] Poteat VP, Russell ST, Dewaele A (2019). Sexual health risk behavior disparities among male and female adolescents using identity and behavior indicators of sexual orientation. Arch Sex Behav.

[4] Centers for Disease Control and Prevention. STDs in Adolescents and Young Adults. 2017. https://www.cdc.gov/std/stats17/adolescents.htm. Accessed 19 Jan 2020.

[5] Centers for Disease Control and Prevention. Sexually transmitted diseases. Washington: Centers for Disease Control and Prevention. 2014. http://www.cdc.gov/std//STDFact-Chlamydia.htm. Accessed 19 Jan 2020.

[6] Flowers P, Barber T, BSocStud, HW, Nelson M, Hedge B, Fakoya A, Sullivan AK. Review of the evidence for the UK national guidelines on safer sex advice. The Clinical Effectiveness Group of the British Association for Sexual Health and HIV (BASHH) and the British HIV Association (BHIVA). 2012. Accessed 19 Jan 2020. https://www.semanticscholar.org/paper/Review-of-the-evidence-for-the-UK-national-on-safer-Flowers-Barber/56882c62104fe07197cea5219930f11b25088dcf

[7] Sully EA, Atuyambe L, Bukenya J, Whitehead HS, Blades N, Bankole A (2018). Estimating abortion incidence among adolescents and differences in postabortion care by age: a cross-sectional study of postabortion care patients in Uganda. Contraception..

[8] Moni SA, Nair MK, Devi RS (2013). Pregnancy among unmarried adolescents and young adults. J Obstetrics Gynecol India.

[9] SDG Indicators.2017. Global Database. https://unstats.un.org/sdgs/indicators/database. Accessed 19 Jan 2020.

[10] Sharifi M, Arman S, Abdoli S, Fard AH, Kohan S (2016). Iranian parents’ experiences about children sexual training: control, restriction and education. Int J Med Res Health Sci.

[11] Morris JL, Rushwan H (2015). Adolescent sexual and reproductive health: the global challenges. Int J Gynecol Obstet.

[12] Darroch JE et al. Research Gaps in Adolescent Sexual and Reproductive Health, New York: Guttmacher Institute. 2016. https://www.guttmacher.org/report/research-gaps-in-sexual-and-reproductivehealth._). Accessed 19 Jan 2020.

[13] Vakilian K, Mousavi SA, Keramat A (2014). Estimation of sexual behavior in the 18-to-24-years-old Iranian youth based on a crosswise model study. BMC Research Notes.

[14] WHO. The global strategy for women’s, children’s and adolescents’ health (2016-2030). 2016. https://www.who.int/life-course/publications/global-strategy-2016-2030/en/. Accessed 19 Jan 2020.

[15] Tanabe M, Schlecht J, Manohar S, Women's Refugee Commission, 2011. Adolescent sexual and reproductive health programs in humanitarian settings: an in-depth look at family planning services. New York: Women's Refugee Commission. Accessed 19 Jan 2020. https://www.unfpa.org/sites/default/files/resource-pdf/AAASRH_good_practice_documentation_English_FINAL.pdf.

[16] Gordon C. Sexuality and Student Health: Access to Sexual and Reproductive Health Resources and Information at American Univeristy. Washington, DC: Master’s Thesis, College of Arts and Sciences, American University; 2012.

[17] Vogel JP, Pileggi-Castro C, Chandra-Mouli V, Pileggi VN, Souza JP, Chou D, Say L (2015). Millennium development goal 5 and adolescents: looking back, moving forward. Arch Dis Child.

[18] Streubert HJ, Carpenter DR. Qualitative research in nursing: advancing the humanistic imperative. 5th ed, Philadelphia: Lippincott Williams & Wilkins; 2011. pp. 468.

[19] UNICEF. The State of the World’s Children 2011: Adolescence: an age of opportunity. 2011. https://www.unicef.org/sowc2011/pdfs/SOWC-2011-Main-Report_EN_02092011.pdf. Accessed 19 Jan 2020.

[20] Bostani Khalesi Z, Ghanbari KA (2015). Understanding and experiencing married women of reproductive age about the importance of sexual health education: a content analysis study. Iran J Obstet Gynecol Infertility.

[21] Mirzaii Nagmabadi K, Babazadeh R, Shariati M, Mousavi SA (2014). Iranian adolescent girls and sexual and reproductive health information and services: a qualitative study. Iran J Obstet Gynecol Infertility.

[22] Mudzongo CC. Longitudinal Analyses of the Sexual and Reproductive Health Knowledge and Parent-Adolescent Communication of At-Risk Adolescents (Doctoral dissertation, North Dakota State University).

[23] World Health Organization. Global accelerated action for the health of adolescents (AA-HA!): guidance to support country implementation. World Health Organization. 2017. Accessed 19 Jan 2020. https://apps.who.int/iris/bitstream/handle/10665/255415/9789241512343-eng.pdf?sequence=1

[24] World Health Organization. Adolescent pregnancy. World Health Organization. 2018. http://www.who.int/en/news-room/factsheets/detail/adolescent-pregnancy. Accessed 19 Jan 2020.

